# Scaphoid fractures with unusual presentations: a case series

**DOI:** 10.4076/1757-1626-2-7220

**Published:** 2009-07-23

**Authors:** Sandesh Pandit, Dennis Y Wen

**Affiliations:** Department of Family and Community Medicine, University of MissouriMA 303 Medical Sciences Building, Columbia, MO 65212USA

## Abstract

Despite the high incidence of scaphoid fractures, their diagnosis and treatment is often delayed which can lead to complications such as non-union, avascular necrosis, and future arthritis. We present three cases with non-classical mechanisms of injury, leading to a delayed diagnosis in all three cases. Our cases serve to emphasize the importance of a high index of suspicion for the possibility of scaphoid fractures in order to avoid these potential complications.

## Introduction

Carpal scaphoid fractures occur fairly frequently. The most commonly described mechanism of injury is a fall on an extended wrist, which creates an axial as well as extension force to the scaphoid bone. We describe three cases of scaphoid fractures which had occurred with less common injury mechanisms, leading to delayed treatment in all three cases. A brief discussion concerning the diagnosis and treatment of scaphoid fractures follows, specifically focusing on management issues when diagnosis has been delayed.

## Case presentations

### Case report 1

A 39 year-old right-handed Caucasian female of American nationality, nurse by occupation, presented to clinic approximately one year after injuring her left wrist. She had been participating in medieval jousting and while holding a sword in her hand, an opponent caused her to hyperpronate her wrist without any direct impact to the wrist or hand itself. She had radiographs taken shortly thereafter, which were apparently negative, and despite symptomatic treatment, she continued to have diffuse radial-sided wrist pain.

**Figure 1.**
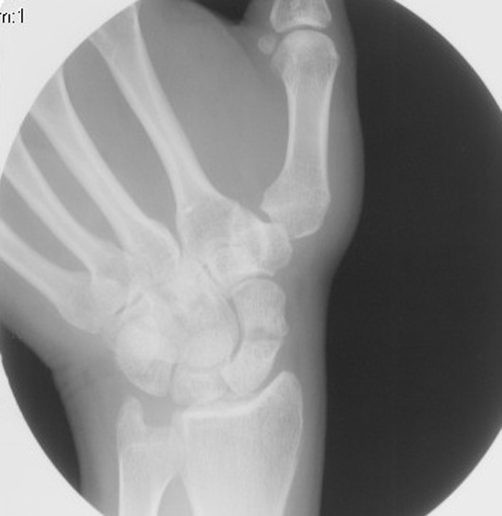
Radiograph of Case #1. Transverse fracture through waist of scaphoid occurring one year previously. No displacement or avascular necrosis is evident.

Her examination in clinic one year after the injury revealed no visible swelling. Passive extension and flexion produced radial-sided pain. There was no tenderness to palpation of the waist of the scaphoid in the snuff box, or over the distal pole of the scaphoid on the palmar side. Repeat radiographs in clinic showed a non-displaced transverse fracture through the waist of the scaphoid without evidence of avascular necrosis (AVN) (Figure 1). MRI was obtained which confirmed the fracture without AVN.

She was treated with prolonged splinting for 6 months along with use of an electromagnetic bone stimulator without complete healing, after which time only symptomatic treatment was given.

### Case report 2

A 20 year-old right-handed Caucasian male of American nationality, who used to deliver water containers, presented to clinic five years after injuring his left wrist. While playing soccer he was hit on the left hand with the ball, causing a forced palmar-flexion of the wrist. He had immediate pain, but was told his radiograph at the time was negative. He wore a removable splint for a few weeks with some initial improvement in symptoms. Subsequently, he had intermittent radial wrist pain, which worsened over time.

Examination of his wrist in clinic 5 years after the injury revealed no visible swelling. His motion was restricted in both flexion and extension. The waist of the scaphoid was non-tender, but the proximal pole near the scapho-lunate junction was tender. Repeat radiographs in clinic showed a non-displaced transverse fracture in the proximal portion of the scaphoid with sclerosis and lucency of the proximal fragment, suggestive of AVN.

Due to the chronicity of the fracture and the associated AVN, internal fixation along with vascularized bone grafting was performed with subsequent adequate healing.

### Case report 3

A 16 year-old right-handed Caucasian male of American nationality presented to clinic one month after the onset of right wrist pain. He had been wrestling with a friend and a few minutes afterwards noticed that his right wrist had severe diffuse pain, encompassing the radial side, the ulnar side, the palmar side, and dorsal side. He recalled no specific trauma other than his friend rolling onto his wrist and forearm. He felt no immediate pain at the time of this rolling incident.

**Figure 2.**
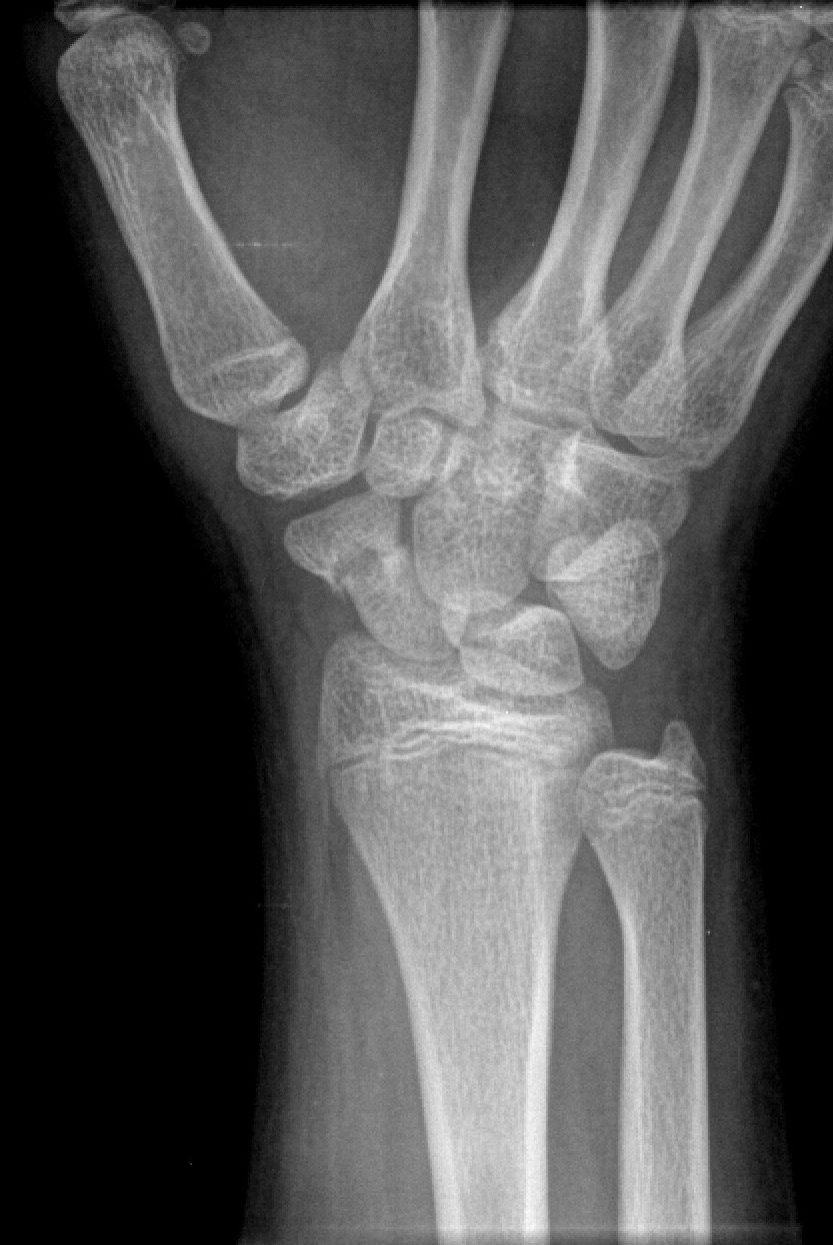
Radiograph of Case #3. Fracture through scaphoid located just distal to waist with mild displacement. Slight widening of the scapholunate interval is also noted. No avascular necrosis is evident.

Examination revealed diffuse swelling over the mid-dorsal and volar regions of the wrist. His wrist was diffusely tender along the dorsoradial side, including the waist of the scaphoid. His range-of-motion was close to the opposite wrist with only mild discomfort at the extreme ranges. His radiograph in clinic showed a minimally displaced transverse fracture at the junction of the distal and middle thirds of the scaphoid with slight widening of the scapholunate interval (Figure 2). He was treated surgically with internal fixation and bone grafting.

## Discussion

Diagnosis and treatment of scaphoid fractures has been fraught with uncertainty and fear for ages. Generations of medical students have learned, and are still learning, that tenderness in the anatomical snuffbox is a cardinal sign of a scaphoid fracture, and that the typical mechanism of injury is a fall on an outstretched hand. However, there have been cases such as ours who neither have the cardinal sign of anatomical snuff box tenderness, nor had they occurred with a fall, and yet they can produce the known complications including nonunion.

Although falls onto outstretched hands are the most commonly described mechanism of scaphoid fractures, other mechanisms have been described including forced palmar flexion of the wrist and axial loading of the wrist with the hand in a fisted position [1]. Although this common wrist fracture has undergone much scrutiny, we have not found mention of cases involving a hyperpronation mechanism, which occurred with our Case #1. Kozin examined several cadaveric studies using varying injury mechanisms and loading conditions [2]. He concluded that the mechanism of injury does not negate the possibility of a scaphoid fracture, and that the heightened suspicion of the physician still remains the most important clue for diagnosing scaphoid fractures.

Avascular necrosis occurs in an estimated 13 to 50% of the scaphoid fractures and the incidence may be even higher in fractures involving the proximal pole [3]. The reason for this high incidence of AVN in cases of proximal pole fractures is thought to be due to the tenuous blood supply of the scaphoid. The blood supply to the scaphoid can be divided into extraosseous and intraosseous sources [4]. The extraosseous blood supply is primarily derived from a branch of the radial artery, the artery to the distal ridge of the scaphoid. The branches of this vessel enter the scaphoid through a foramen at the dorsal ridge at the level of the waist. These vessels then run proximally and palmarly within the medullary chamber, forming the intraosseous supply to the proximal pole. Since vascularity of the proximal pole is limited and dependent on intraosseous flow, acute proximal pole fractures have a potentially prolonged healing period, averaging 3 to 6 months, and there is higher incidence of non-union.

Tenderness in the anatomical snuffbox is often described as a classical sign of scaphoid fracture with a reported sensitivity of 90% but a low specificity of 40% [5]. Our first two Cases had no snuffbox tenderness, although the proximal pole was tender in Case #2. The scaphoid compression test is performed by grasping the thumb and applying an axially directed compressive force along the line of the thumb metacarpal with wrist pain indicating a positive result [6]. Chen, in his series of 52 traumatized wrists with snuffbox tenderness, reported high sensitivity and specificity of this compression test for scaphoid fractures [7]. However, another study demonstrated poor predictive value of this compression test for identifying scaphoid fractures [8].

Acute scaphoid fractures can often be missed on initial plain radiographs, with reported sensitivities ranging from 84% to 98% [9]. When clinical suspicion of a scaphoid fracture is high and plain films are negative, the traditional recommendation is for these patients to be immobilized in a thumb spica splint or cast with repeat radiographs after about two weeks. There is no consensus concerning whether a short-arm or long-arm splint or cast is preferable. In a comparative study of short and long arm casting, it was concluded that non-displaced fractures heal well regardless of the type of the cast used [10]. Another randomized prospective trial found that immobilization of the thumb did not improve the outcome for non-displaced fractures [11], despite the traditional view that thumb immobilization was necessary.

Recently, the traditional approach of casting suspected scaphoid fractures with negative radiographs has been criticized, citing that this approach would result in approximately 75-90% of patients being immobilized unnecessarily for a week or more [12]. Alternative imaging modalities for diagnosis include bone scans and MRIs. A bone scan reportedly shows focally increased uptake within 72 hours and is very sensitive to detect a fracture, but may not be extremely specific [12].

MRI has been reported to have 95 to 100% sensitivity and almost 100% specificity for scaphoid fractures [3]. Additional advantages of an MRI include its ability to identify other potential causes of wrist pain when a scaphoid fracture is not present, as well as its potential to assess vascularity of the proximal scaphoid pole when a fracture is present [3]. Despite its superiority to other modalities, routine MRI is still controversial because of its cost and availability. Brooks and Cicuttini [13] performed a randomized controlled trial to investigate the cost effectiveness of adding MRI to the usual management of suspected scaphoid fractures and concluded that when productivity losses due to immobilization are considered, then MRI may be considered cost effective [13]. An MRI tailored to evaluate suspected carpal fracture has been suggested to be equivalent in cost to the traditional approach of immobilization followed by repeat plain films [12,13,14]. If there is urgency to obtain an early definitive diagnosis, an MRI can be obtained for definitive diagnosis, but otherwise immobilization followed by repeat plain films in 7-10 days is still considered an acceptable approach.

Although acute non-displaced fractures have traditionally been treated non-operatively in a cast with a reported 90% union rate, a growing body of literature suggests that greater union rates approaching 95% could be obtained with immediate operative management, which can lead to earlier healing, better range of motion, and an earlier return to work [15]. However, a recent randomized controlled trial comparing the long term results of operative fixation of acute scaphoid fractures with that of non-operative treatment did not demonstrate long-term benefits of surgery [16]. However, short-term differences between groups were not reported. If non-operative treatment for non-displaced or minimally displaced fractures is elected, the recommended duration of immobilization varies with the anatomical area injured, with distal pole fractures requiring immobilization for 4 to 6 weeks, waist fractures for 10 to 12 weeks, and proximal pole fractures for 12 to 20 weeks [12].

Scaphoid fractures with displacement of more than 1 mm, associated AVN, established non-union, or associated scapholunate dissociation, may require operative intervention [12]. Therefore, surgery was recommended for Case #2 who had established AVN at presentation, and for Case #3 who had mild fracture displacement and possible scapholunate dissociation.

When a fracture is missed in the acute stage and the patient presents with an existing non- union such as two of our patients (Cases #1 and #2), the treatment becomes more challenging especially if AVN is present. Scaphoid non-unions can notoriously progress to carpal collapse and degenerative arthritis [14]. Non-operative treatment for nondisplaced scaphoid non-unions can involve very prolonged periods of cast immobilization (4 to 6 months) with or without electrical or ultrasound bone stimulation, which can negatively impact quality of life [17]. Case #1 had a chronic non-union, but no radiographic evidence of AVN, and so prolonged cast immobilization along with bone stimulation was attempted, which in this case was not successful. For Case #2, who had AVN on his presenting radiographs, surgery was performed successfully.

A variety of surgical techniques have been described for treating scaphoid fractures, with different techniques advocated for different clinical situations [17]. Most surgical series have reported union rates of about 90% with these various procedures [18]. The most commonly cited reason for failed surgical union has been AVN of the proximal pole with reported union rates from 40 to 67% with non-vascularised bone grafts [18]. Vascularised bone grafting has recently gained popularity [19]. In cases of progressive arthrosis salvage procedures like intercarpal fusion, proximal row carpectomy, scaphoid excision with four corner arthrodesis, total wrist arthroplasty, or total carpal fusion may be necessary [18].

In conclusion, we have presented three cases of scaphoid fractures with non-classical injury mechanisms. Our cases also highlight the point that not all patients with scaphoid fractures exhibit snuffbox tenderness. Physicians should have a high index of suspicion for this injury, even without the typical history of falling onto an out-stretched wrist, or without the typical snuffbox tenderness, since missing or delaying the diagnosis and subsequent treatment can lead to chronic non-unions and subsequent degenerative arthritis along with their associated disabilities.

## Authors’ information

Sandesh Pandit is resident in the Department of Family and Community Medicine at the University of Missouri School of Medicine, Columbia. He received his medical degree from Institute of Medicine; Kathmandu, Nepal.

Dennis Y. Wen, is associate professor in the Department of Family and Community Medicine at the University of Missouri School of Medicine, Columbia. He received his medical degree from East Carolina University School of Medicine; Greenville, N.C. Wen completed a family medicine residency and a sports medicine fellowship at the University of California, Los Angeles.

## Abbreviations

AVN: avascular necrosis
MRI: magnetic resonance imaging
